# Pentose bisphosphate pathway can act in central metabolism for nucleoside-dependent growth of *Thermococcus kodakarensis* strains

**DOI:** 10.1128/aem.01712-25

**Published:** 2025-10-29

**Authors:** Tetsu Nishida, Yangzi Chen, Takehiro Azuma, Izumi Orita, Toshiaki Fukui

**Affiliations:** 1 School of Life Science and Technology, Institute of Science Tokyo 163706 , Yokohama, Japan; Michigan State University, East Lansing, Michigan, USA

**Keywords:** hyperhermophile, Thermococcales, *Thermococcus kodakarensis*, pentose bisphosphate pathway, RuBisCO, nucleoside transporter, archaea

## Abstract

A laboratory-evolved strain of *Thermococcus kodakarensis* was found to grow in a nutrient-rich medium without elemental sulfur (S^0^) or pyruvate, unlike the wild type. The growth of the evolved strain was stimulated by nucleoside supplementation, and the cells consumed nucleosides, accompanied by accumulation of nucleobases in the medium, indicating utilization of the ribose moiety by the cells for growth. The H_2_/CO_2_ production ratio was approximately 2:1, and the total amount of acetate and alanine produced was consistent with the amount of nucleosides consumed. These observations support the assimilation of the ribose moiety from nucleosides through the pentose bisphosphate (PBP) pathway employing the CO_2_-fixing form III RuBisCO. The evolved strain utilized pyruvate derived from nucleosides as both an energy source and an acceptor of an amino group to sustain peptides/amino acids catabolism even in the absence of S^0^ and pyruvate, of which the ability has been lacking in the wild-type strain. Whole-genome re-sequencing of the evolved strain identified mutations both upstream of the RuBisCO gene and within the coding region of a substrate-binding periplasmic component of a putative nucleoside-transporter subunit (NupD) with a domain organization distinct from previously characterized components. The mutation in the upstream region of the RuBisCO gene led to the upregulated expression, and those in *nupD* possibly enhanced nucleoside uptake by the NupD-containing transporter. The strain into which these mutations were reconstituted was able to grow in the nucleoside-supplemented medium without S^0^ or pyruvate.

**IMPORTANCE:**

*Thermococcus kodakarensis* usually requires S^0^ or pyruvate for the processing of the amino group when grown on peptides/amino acids. This study demonstrated that adaptive laboratory evolution enabled this archaeon to utilize nucleosides as a growth substrate via the carboxylating PBP pathway. Two mutations, one leading to higher expression of the CO_2_-fixing RuBisCO and the other enhancing nucleoside uptake, were found to be key modifications for the nucleoside-dependent growth. The results also indicated that NupDABC, containing NupD with a unique primary structural property, was the archaeal nucleoside-specific transporter. These findings demonstrated that the PBP pathway is not only a nucleoside salvage module but also potentially acts as a central metabolic module supporting cell growth when nucleosides are available. This expands our understanding of metabolic flexibility in hyperthermophilic archaea and suggests potential applications of the carboxylating PBP pathway in biotechnological processes involving CO_2_ fixation.

## INTRODUCTION

*Thermococcus kodakarensis*, a member of the archaeal order Thermococcales, is a hyperthermophile that can grow at temperatures above 90°C (1–3) and has been a well-studied species owing to the availability of practical genetic tools (4–6). Many studies on carbon and energy metabolisms in the Thermococcales archaea have identified several pathways distinct from those in bacteria and eukarya, such as the modified Embden-Meyerhof pathway (7, 8).

*T. kodakarensis* preferentially grows on peptides/amino acids as carbon and energy sources (9), where the initial reaction of the amino acid metabolism is the transfer of the amino group to 2-oxoglutarate (2OG), forming glutamate (Glu) (10–13). In the presence of elemental sulfur (S^0^), 2OG is regenerated through oxidative deamination of Glu by NADP^+^-dependent glutamate dehydrogenase (14). The 2-oxoacids formed by the deamination of amino acids are oxidized to their corresponding acids, producing ATP and reduced ferredoxin. The reduced ferredoxin is then used to reduce NADP^+^ to NADPH, which is regenerated to the oxidized form by donating electrons for the reduction of S^0^ to H_2_S (14). In the absence of S^0^, exogenous pyruvate or that generated from supplemented starch can act as an acceptor of the α-amino group from Glu. This transamination regenerates 2OG from Glu and produces Ala as one of the end products during growth (15). Part of pyruvate is oxidized along with other amino acid-derived 2-oxoacids via ferredoxin-dependent oxidative decarboxylation. In contrast to S^0^-dependent conditions, the reduced ferredoxin is re-oxidized by membrane-bound hydrogenase using protons as the terminal electron acceptor with concomitant evolution of hydrogen (16). These metabolic profiles suggest that *T. kodakarensis* requires a sufficient amount of S^0^ or pyruvate when growing on amino acids.

The pentose phosphate pathway, consisting of oxidative and non-oxidative branches, generates NADPH and pentose phosphates such as ribose-5-phosphate (R5P) and erythrose-4-phosphate (E4P) for nucleotide and aromatic compound biosynthesis (17). Archaeal genome analyses interestingly indicated that many species, with the exception of halophilic archaea, lack the oxidative branch of the pentose phosphate pathway (18). In addition, most species belonging to the phyla Methanobacteriota and Thermoproteota, with the exception of the genera *Methanocaldococcus* and *Methanococcus*, lack transaldolase and ribulose-5-phosphate (Ru5P)-3-epimerase. This incomplete non-oxidative branch is incapable of degrading the pentose moieties as well as synthesizing E4P (18). In this kind of archaea, the ribulose monophosphate pathway (19) and the pentose bisphosphate (PBP) pathway (20–22) have been proposed to involve the conversion of sugar phosphates instead of the pentose phosphate pathway.

The ribulose monophosphate pathway, originally discovered in methylotrophic bacteria that can utilize C_1_ compounds as growth substrates (23), has also been identified in hyperthermophilic archaea, including *Pyrococcus horikoshii* and *T. kodakarensis*. In these archaea, a fusion of the key enzymes of this pathway, 3-hexulose-6-phosphate synthase and 6-phospho-3-hexuloisomerase, mediates the cleavage reaction of fructose-6-phosphate to Ru5P and formaldehyde to provide R5P for nucleotide synthesis (19, 24). The PBP pathway contributes to the degradation of the pentose phosphate in some archaea. The whole-genome analyses also clarified the presence of form III ribulose-1,5-bisphosphate carboxylase/oxygenase (RuBisCO), consisting solely of a large subunit, in a wide lineage of archaea (25, 26). Although the metabolic significance of the form III RuBisCO in these archaea, not possessing the Calvin-Benson-Bassham cycle, had been unknown since its discovery, recent studies revealed that the form III RuBisCO functions in the PBP pathway for nucleoside salvage (20–22). In *T. kodakarensis*, the newly identified ribose-1,5-bisphosphate (R15P) isomerase catalyzes the conversion of R15P, produced from nucleoside monophosphates or ribose monophosphates, to ribulose-1,5-bisphosphate (RuBP) which is a substrate of RuBisCO. RuBisCO catalyzes the formation of two molecules of 3-phosphoglycerate (3PGA) from RuBP with CO_2_ fixation, and 3PGA then enters glycolysis/gluconeogenesis. This pathway is now proposed to be the carboxylating PBP pathway (20–22). Meanwhile, in halophilic archaea, the non-carboxylating PBP pathway operates to convert the nucleoside-derived RuBP to ribulose-1-phosphate (Ru1P), which is then cleaved by Ru1P aldolase into dihydroxyacetone phosphate and glycolaldehyde (27).

In this study, we isolated a unique mutant strain of *T. kodakarensis* through adaptive evolution that is capable of growing in a nutrient-rich medium containing peptides/amino acids even in the absence of S^0^ or pyruvate/starch, despite the strict requirement for S^0^ or pyruvate for the parent strain to grow under such conditions. Further detailed analyses of this mutant demonstrated the potential significance of the carboxylating PBP pathway as a metabolic process supporting the nucleoside-dependent growth of this archaeon and also identified an archaeal transporter responsible for nucleoside uptake.

## MATERIALS AND METHODS

### Strains and culture conditions

The strains and plasmids used in this study are listed in Table 1. *Escherichia coli* strains were usually cultivated at 37°C in a Lysogeny broth (LB) medium. 100 µg/mL ampicillin was added to the medium when necessary. *T. kodakarensis* KUW1 (Δ*pyrF* Δ*trpE*) (5) and its derivative strains were cultivated under anaerobic conditions at 85°C in nutrient-rich ASW-YT-based media, which were composed of a 1.25-fold dilution of artificial seawater (0.8 × ASW) (9), 10 g/L of yeast extract (Nacalai Tesque, Kyoto, Japan), and 5.0 g/L of tryptone (Nacalai Tesque). When necessary, 2.0 g/L of elemental sulfur (ASW-YT-S^0^), 5.0 g/L of pyruvate (ASW-YT-Pyr), or 5.0 g/L of glycerol (ASW-YT-Glycerol) was added to the medium to support the cell growth. A synthetic medium (ASW-AA-S^0^) was used for several genetic manipulations as described previously (4). A solidified ASW-YT-S^0^ medium was prepared by adding 2 mL/L of polysulfide solution (10 g of Na_2_S·9H_2_O and 3 g of sulfur flowers in 15  mL of H_2_O) and 10 g/L of gellan gum (FUJIFILM Wako Pure Chemical Corporation, Osaka, Japan) to the ASW-YT medium. 7.5  g/L of 5-fluoroorotic acid (5-FOA, Apollo Scientific, Stockport, UK) was supplemented to the solid medium for counter selection. All the anaerobic manipulations were done within a COY anaerobic chamber (COY Lab Products, Grass Lake, MI, USA).

**TABLE 1:** Strains and plasmids in this study^*a*^

Strain or plasmid	Relevant marker	Source
*Escherichia coli* strain		
DH5α	F^–^, Φ80d*lacZ*∆M15, ∆(*lacZYA-argF*)U169, *deoR*, *recA1*, *endA1*, *hsdR17*(r_k_^−^,m_k_^+^), *phoA*, *sup*E44, λ^−^, *thi*-1, *gyr*A96, *relA1*	Takara Bio
*Thermococcus kodakarensis* strains		
KOD1	Wild type	(9)
KUW1	KOD1 derivative; ∆*pyrF*, Δ*trpE* (uracil and tryptophan auxotroph)	(5)
KUW1-*P_csg_-glpFK_Tm_*-G3PDH	KUW derivative; *P_csg_-glpFK_Tm_*::*tk1393*	This study
ALE22	KUW1-*P_csg_-glpFK_Tm_*-G3PDH derivative; evolved for 22 generations in ASW-YT-Glycerol medium	This study
KUW1-∆rbc	KUW1 derivative; ∆*tk2290*	This study
ALE22-∆rbc	ALE22 derivative; ∆*tk2290*	This study
KUW1-∆nupD	KUW1 derivative; ∆*tk0657*	This study
ALE22-∆nupD	ALE22 derivative; ∆*tk0657*	This study
KUW1-rbc_mut_	KUW1 derivative; point mutation (A to G) at −16b from the start codon of *tk2290*	This study
KUW1-NupD_mut_	KUW1 derivative; two-point mutations in *tk0657* (C116T and A349G)	This study
Plasmids		
pUC118	Amp^r^, general cloning vector	Takara Bio
pUD3	pUC118 derivative; *pyrF* marker cassette	(28)
pUD3-up-*P_csg_-glpFK*-down	pUD3 derivative; for insertion of *P_csg_-glpFK_Tm_* into upstream of *tk1393*	This study
pUD3-∆rbc	pUD3 derivative; for deletion of *tk2290*	This study
pUD3-∆nupD	pUD3 derivative; for deletion of *tk0657*	This study
pUD3-rbc-up-mut	pUD3 derivative; for reconstitution of point mutation at upstream of *tk2290* (A to G at −16b from the start codon of *tk2290*)	This study
pUD3-NupD_mut_	pUD3 derivative; for reconstitution of point mutations in *tk0657* (C116T and A349G)	This study
pLC71	*T. kodakarensis-E. coli* shuttle vector, Amp^r^, Km^r^, *trpE_Tk_*, *hmg_Pf_*	(6)
pLCSM	pLC71 derivative; ∆Km^r^ ∆*trpE_Tk_*	This study
pLCSH	pLCSM derivative; ∆*P_pyrF_*::*P_csg_*	This study
pLCSM-rbc	pLCSM derivative; *P_pyrF_*::*tk2290*	This study
pLCSH-rbc	pLCSH derivative; *P_csg_*::*tk2290*	This study

^*a*^ Tk, *T. kodakarensis*; Tm, *T. maritima*; Pf,* P. furiosus*.

### Plasmid construction

DNA manipulations were carried out according to standard procedures. PCRs were performed with KOD DNA polymerase variants purchased from Toyobo (Osaka, Japan). The sequences of oligonucleotide primers used in this study are shown in Table S1.

*T. kodakarensis-E. coli* shuttle vector pLCSM was constructed by removing Km^r^ and *trpE* from pLC71 (6) and inserting a fragment containing NdeI-StuI-SpeI-NheI-SphI restriction sites downstream of the *pyrF* promoter (*P_pyrF_*) region. Another shuttle vector pLCSH was constructed by replacing *P_pyrF_* in pLCSM with the promoter region of the cell surface glycoprotein (*csg*, TK0895) (*P_csg_*). The coding region for RuBisCO (TK2290), amplified from genomic DNA of *T. kodakarensis* as a template, was individually inserted into the downstream region of *P_pyrF_* in pLCSM and *P_csg_* in pLCSH at the NdeI/SpeI sites, to construct RuBisCO-expression vectors pLCSM-rbc and pLCSH-rbc, respectively.

The vector for inserting a fusion of *P_csg_* and a tandem of *glpFK* from *Thermotoga maritima* into the upstream of *tk1393* encoding the large subunit of G3PDH, pUD3-up-*P_csg_-glpFK*-down, was constructed as follows. The fragments of *P_csg_* and a tandem of *glpFK_Tm_* were amplified from genomic DNAs of *T. kodakarensis* and *Thermotoga maritima*, respectively, and pUC118-*P_csg_-glpFK_Tm_* was obtained by inserting these fragments into the HincII site in pUC118. A fragment of 2 kbp around the start codon of *tk1393*, amplified by PCR from the *T. kodakarensis* genomic DNA, was inserted into pUD3 harboring a *pyrF* marker cassette (4, 28) at the SalI restriction site. The resulting plasmid was linearized by inverse PCR at the start codon of *tk1393*. The fragment of *P_csg_-glpFK_Tm_*, prepared from pUC118-*P_csg_-glpFK_Tm_*, was ligated with the linearized vector.

The deletion vectors for the NupD and RuBisCO genes, pUD3-∆nupD and pUD3-∆rbc, were constructed by inserting the respective fragment of the target gene along with the flanking upstream and downstream regions (~800 bp each) into pUD3 and subsequent deletion of the target region by inverse PCR and self-ligation. The vectors for reconstitution of mutations identified in *T. kodakarensis* strain ALE22, pUD3-NupD_mut_ and pUD3-rbc-up-mut, were constructed by inserting the respective fragments harboring the mutated region flanking the upstream and downstream regions (~800 bp each) into pUD3. The resulting vectors were sequenced and confirmed to have no unintended mutations.

### Transformation of *T. kodakarensis*

Transformation of *T. kodakarensis* KUW1 with the pUD3-based vectors and selection of transformants formed by pop-in/pop-out recombination were performed according to the procedure described previously (5). The successful recombination was verified by PCR using appropriate primer sets. Introduction of the shuttle vectors into *T. kodakarensis* was examined as reported previously (29) except for the use of 5 µM lovastatin (Tokyo Chemical Industry, Tokyo, Japan) as the antibiotic. The transformant was isolated using a lovastatin-free ASW-YT-S^0^ solid medium.

### Adaptive laboratory evolution

The strain KUW1-*P_csg_-glpFK_Tm_-g3pdh* was subjected to adaptive laboratory evolution. The cells grown in 100 mL of ASW-YT-Pyr medium for 16 h were harvested and washed with 0.8× ASW, and then resuspended with 100 mL of ASW-YT-Glycerol medium. After the cultivation at 85°C for a week (the 1st generation), all of the cells were again harvested, washed, transferred into 100 mL of the fresh glycerol medium, and cultivated for another week at 85°C (the 2nd generation). After 16 rounds of serial passages, 14 strains were isolated on the ASW-YT-Glycerol solid medium from the cells of the 16th generation. Among the isolated strains capable of growing in the ASW-YT-Glycerol liquid medium, a strain that showed the best growth in the glycerol medium was selected. The selected strain was cultivated in the ASW-YT-Glycerol medium at 85°C for 6 days and subjected to 6 rounds of subculture with inoculation of 1% (vol/vol) culture broth into the fresh glycerol medium, followed by cultivation at 85°C every 3–6 days. After the 6th subculture, one strain, termed ALE22, was isolated by the plate cultivation on the ASW-YT-Glycerol solid medium. The stock of ALE22 was established with the cells grown in ASW-AA-S^0^ liquid medium and then was used for further investigations.

### Determination of growth properties

*T. kodakarensis* cells were precultured in 10 mL of ASW-YT-Pyr for 16 h, and the cells at the stationary phase were inoculated into 8 mL of ASW-YT-based media. The cultivation was performed anaerobically in a test tube tightly capped with a butyl rubber stopper at 85°C in triplicate or duplicate. The optical density at 600 nm (OD_600_) of the broth in the test tube was directly measured by using DEN-600 (Biosan, Riga, Latvia). When necessary, various nucleoside(s) or the related compound(s) were supplemented into ASW-YT medium as follows: 0.1% wt/vol each of inosine and uridine (ASW-YT-IU); 0.1% or 0.2% wt/vol of adenosine, guanosine, inosine, cytidine, or uridine (ASW-YT-U); 0.1% wt/vol of hypoxanthine, inosine-5′-monophosphate, or deoxythymidine.

### Analyses of gas phase and culture supernatant

*T. kodakarensis* strains were precultured in 10 mL of ASW-YT-Pyr at 85°C for 16 h, and 1.2 mL of the broth was inoculated into 120 mL of an ASW-YT-U medium. The gas-capturing cultivation using a 100 mL bottle (40 mL headspace) equipped with a 0.5 L-volume aluminum bag, and determination of concentrations of H_2_ and CO_2_ in the captured gas by gas chromatography was done as reported previously (29).

The supernatant of the culture broth samples was filtered by using a Minisart RC4 (Sartorius, Göttingen, Germany) with a pore size of 0.2 µm to remove particulate contaminants. The supernatant samples were subjected to acid treatment to hydrolyze proteins and peptides as previously described (30), or treated with nuclease and alkaline phosphatase to degrade nucleic acids and nucleotides to nucleosides. The enzymatic treatment was done in 200 µL of 0.1 M trimethylamine-acetic acid buffer (pH 5.8) containing 0.1 µL of the supernatant, 20 U of nuclease P1 (New England Biolabs, Ipswich, MA, USA), and 0.4 U of alkaline phosphatase (Shrimp) (Takara Bio Inc., Shiga, Japan) at 37°C for 3 h. The sample was dried completely by centrifugal evaporation at 55°C for 2–3 h and dissolved in 20 µL of 90% acetonitrile. The amino acids in the acid-treated sample, and nucleosides and nucleobases in the enzyme-treated sample were determined by an LC-MS/MS system consisting of Nexera UHPLC (Shimadzu, Kyoto, Japan) equipped with Discovery HS F5 (3 µm) HPLC Column (Supelco, Bellefonte, PA, USA) and LCMS-8050 (Shimadzu) with a non-ion-pair method in the LC/MS/MS Method Package for Primary Metabolites Ver.3 (Shimadzu). A binary gradient with 0.1% formic acid in water and 0.1% formic acid in acetonitrile was applied according to the method package provided by the manufacturer. Quantification of acetic acid was done with Nexera X2 Multi-System (organic acid) equipped with a Shim-pack SCR-102H column and a conductivity detector (Shimadzu). A commercially available mobile phase reagent set (Shimadzu) for organic acid analysis was used.

### qRT-PCR and Native-PAGE

The cells of *T. kodakarensis* strains at the stationary phase grown in an ASW-YT-Pyr medium were harvested, and the total RNA was extracted by using RNeasy Kits for RNA Purification (Qiagen, Hilden, Germany). Reverse transcription and qRT-PCR on the extracted RNA were conducted by ReverTra Ace (Toyobo) and KOD SYBR (Toyobo), respectively. The sequences of the primers for qRT-PCR are listed in Table S1. Threshold cycle (Ct) values for *tk0308,* which encodes a translation elongation factor and is constitutively expressed under various culture conditions, were used to normalize the transcription level.

A cell-free extract from the harvested cells was prepared by sonication in 100 mM Tris-HCl buffer (pH 7.5) and successive centrifugation at 18,000 *× g* for 10 min. Protein concentration of the soluble fraction was determined by the Bradford method using Protein Assay CBB Solution (Nacalai Tesque). The soluble fractions containing 5 µg protein and a size marker (WSE-7016 EzStandard Native, ATTO, Tokyo, Japan) were subjected to Native-PAGE with an 8% polyacrylamide gel, and stained with Coomassie Brilliant Blue (CBB-1000, BIO CRAFT, Tokyo, Japan).

### Genome re-sequencing analysis

DNA library construction and sequencing of the genomic DNA of the strains KUW1 and ALE22 were performed by Novogene (Beijing, China) using the Illumina Novaseq 6000 platform. The mapping coverage (≥1×) and average depth for KUW1 were 99.95% and 1856.24, and those for ALE22 were 99.91% and 2004.97, respectively.

## RESULTS

### Isolation of a *T. kodakarensis* mutant capable of growth without elemental sulfur and pyruvate through adaptive laboratory evolution

**Fig 1:**
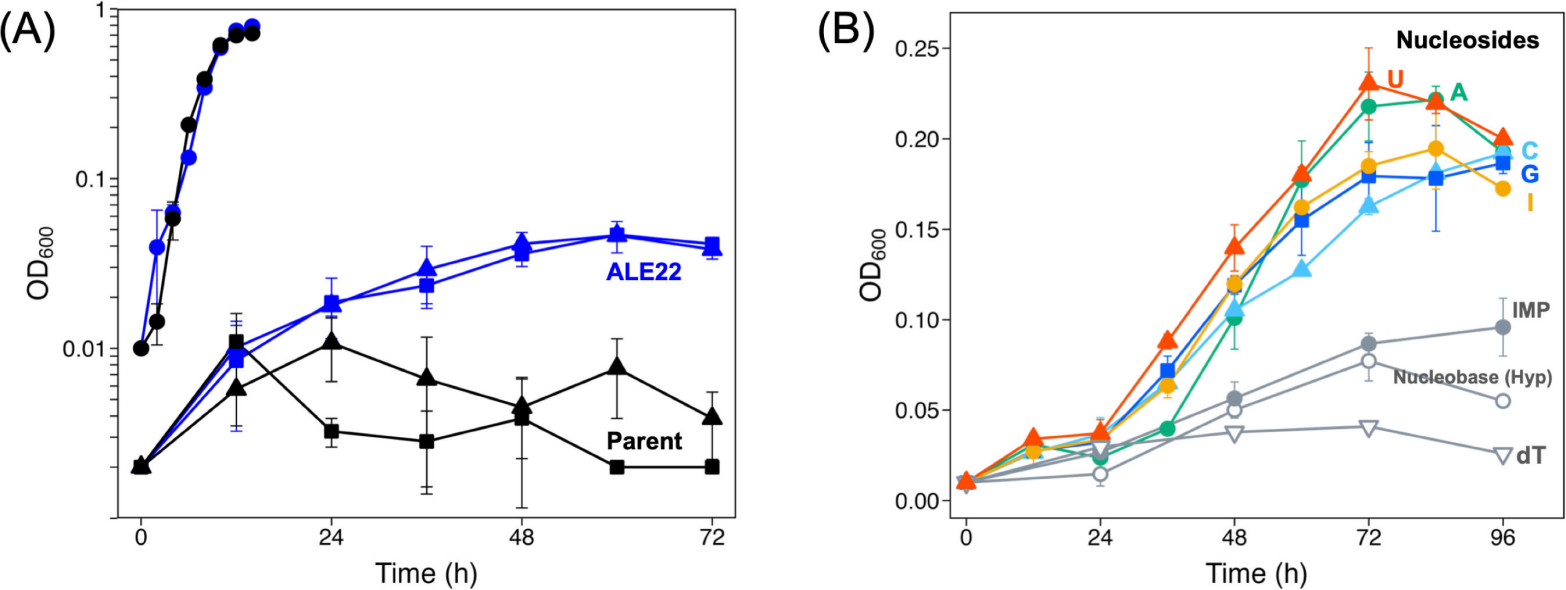
(**A**) Growth properties of the parent strain KUW1-P*_csg_*-glpFK*_Tm_*-G3PDH (black) and ALE22 (blue) in a nutrient-rich ASW-YT media supplemented with 0.5% (wt/vol) of pyruvate (circles), 0.5% (wt/vol) glycerol (triangles), or none (squares). (**B**) Growth properties of ALE22 in ASW-YT media supplemented with 0.1% (wt/vol) of adenosine (green circles), guanosine (blue squares), inosine (yellow circles), cytidine (sky-blue triangles), uridine (orange triangles), thymidine (open gray inverted triangles), hypoxanthine (open gray circles), or inosine-5′-monophosphate (closed gray circles). The cells were anaerobically cultivated in the 8 mL medium using a test tube at 85°C for 96 h. Error bars represent standard deviations of three or two independent culture experiments.

*T. kodakarensis* is unable to grow on glycerol in the absence of S^0^ and pyruvate, although this strain possesses the *tk1393–tk1392–tk1391* gene cluster encoding an anaerobic glycerol-3-phosphate dehydrogenase (G3PDH), which is expected to support the growth by producing dihydroxyacetone phosphate, a glycolytic intermediate. Focusing on the bioproduction of hydrogen from waste glycerol, we attempted to engineer *T. kodakarensis* to confer glycerol-assimilating ability. As the growth deficiency on glycerol might be attributed to a lack of machinery for glycerol uptake and/or phosphorylation, the heterologous genes from *Thermotoga maritima* encoding glycerol facilitator (*glpF_Tm_*) and glycerol kinase (*glpK_Tm_*) were inserted into the locus upstream of *tk1393* encoding the large subunit of G3PDH, thereby establishing a metabolic link from exogenous glycerol to glycerol-3-phosphate. Unfortunately, the constructed strain still showed no growth in ASW-YT-glycerol medium. We then conducted adaptive laboratory evolution of the engineered strain by serial passages in the ASW-YT-glycerol medium, and one strain was isolated after a total of 22 rounds of passages, which comprised 16 rounds of transferring all the cells into fresh medium followed by 6 rounds of serial subcultures. The isolated strain termed ALE22 indeed grew slowly in the glycerol medium, while we noticed that the strain showed similar growth in the ASW-YT medium containing neither pyruvate nor glycerol when compared with that in the medium containing glycerol (Fig. 1A). The evolved strain was presumed to utilize some components in the nutrient-rich medium for growth.

### Consumption of ribonucleosides by the evolved strain

Since the complex ASW-YT medium contains a large amount of RNA that is the second most abundant component in yeast extract after proteins (31, 32), the effects of supplementation of various RNA-related compounds on the growth of ALE22 were investigated. As shown in Fig. 1B, the supplementation of five types of ribonucleosides—adenosine, guanosine, inosine, cytidine, and uridine—resulted in an increase in OD_600_ after 72 h. In contrast, the addition of a nucleobase (hypoxanthine), a deoxyribonucleoside (deoxythymidine), or a nucleotide (inosine-5’-monophosphate) had no effect on the growth. These results strongly suggested that ALE22 utilized ribonucleosides as growth substrates.

The concentration of ribonucleic acids and related compounds in the ASW-YT medium was determined by LC-MS/MS analysis. RNA and ribonucleotides in the culture supernatant were hydrolyzed by RNase and dephosphorylated by alkaline phosphatase and then quantified as nucleosides. The free nucleosides in the culture supernatant were thus quantified from the samples without enzymatic treatment, whereas the total amount of free nucleosides, RNA, and ribonucleotides in the culture supernatant was determined from the enzymatically treated samples. In the ASW-YT medium incubated at 85°C for 96 h without inoculation, the concentration of free nucleosides was 0.50 mmol/L-culture. The total concentration of nucleosides was determined to be 1.97 mmol/L-culture after the enzymatic treatment, indicating the presence of 1.47 mmol/L-culture equivalent of RNA and nucleotides in the medium. After the cultivation of ALE22 at 85°C for 96 h, almost no free nucleosides were detected without the enzymatic treatment, and 0.77 mmol/L-culture of total nucleosides was detected in the treated supernatant. Thus, the cells consumed all 0.50 mmol/L-culture of the free nucleosides and 0.70 mmol/L-culture of the nucleosides derived from RNA or nucleotides in ASW-YT.

### Involvement of the PBP pathway in the nucleoside-dependent growth

**Fig 2:**
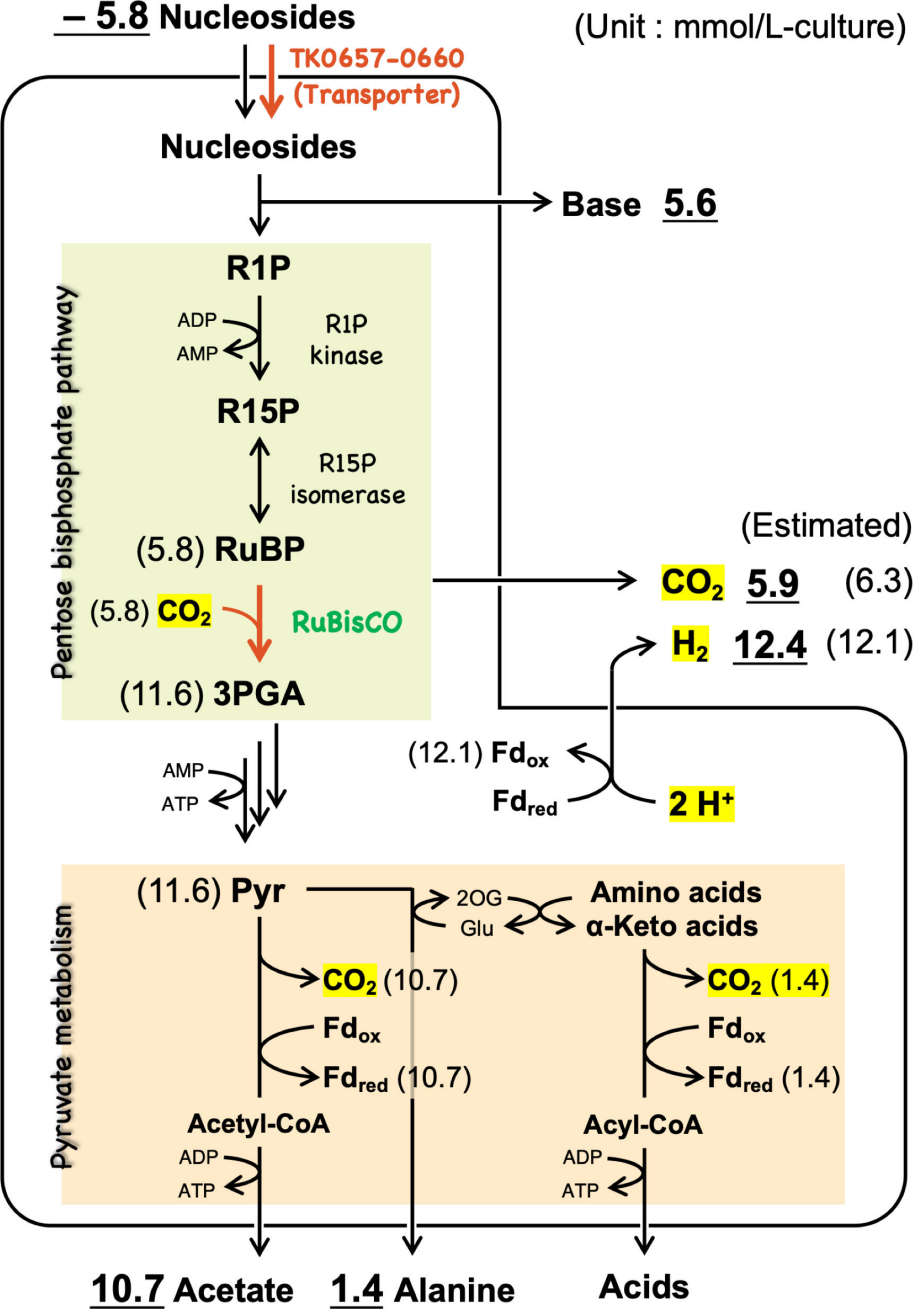
Mass balance profile in ALE22 grown in an ASW-YT medium supplemented with 0.1% (wt/vol) (4.1 mM) of uridine at 85°C for 96 h with gas capturing. The values with underlines represent concentrations of the metabolites determined by LC-MS/MS or HPLC analysis of the culture supernatant, or GC analysis of the evolved gas. The values in parentheses represent the estimated concentrations based on actual measurements for nucleosides, acetate, and alanine.

In *T. kodakarensis*, the ribose moiety in ribonucleotides is converted to 3-phosphoglycerate (3PGA) via the PBP pathway and then enters the modified Embden-Meyerhof pathway. We thus considered that pyruvate derived from the ribose moiety through the PBP pathway could act as an acceptor of the amino group in the catabolism of amino acids (alanine-forming), as well as become a source to conserve reducing equivalents and ATP through oxidative catabolism (acetate-forming) to support the growth (15). It has been reported that the *T. kodakarensis* recombinant lacking H_2_-uptake cytosolic hydrogenase evolved H_2_ and CO_2_ with the ratio of 1:1 when grown in ASW-YT-Pyr medium containing amino acids and pyruvate (30). This ratio was predicted to be changed when the nucleoside-derived ribose moiety was metabolized through the carboxylating PBP pathway. Here, the consumption of nucleosides and production of H_2_, CO_2_, acetate, and alanine were determined for the strain ALE22 cultivated with gas capturing in ASW-YT medium supplemented with 4.1 mM uridine. During the cultivation at 85°C for 96 h, the cells consumed 5.8 mmol/L-culture of the nucleosides and produced 10.7 mmol/L-culture of acetate and 1.4 mmol/L-culture of alanine (Fig. 2). When all the ribose moieties in nucleosides were converted through the PBP pathway, 11.6 mmol/L-culture of pyruvate was estimated to have been formed from the consumed nucleosides and then converted. The sum of acetate and alanine accumulated in the medium was 12.1 mmol/L-culture, which was in good agreement with the estimated formation of the nucleoside-derived pyruvate. As the conversion of ribose to two molecules of pyruvate through the PBP pathway contains the fixation of CO₂ mediated by RuBisCO, the theoretical ratio of the evolved H_2_ and CO_2_ is expected to become 2:1 during the nucleoside-dependent growth. The strain ALE22 evolved 12.4 mmol/L-culture and 5.9 mmol/L-culture of H_2_ and CO_2_, respectively. The actual H_2_/CO_2_ ratio was 2.1:1, which was also consistent with the involvement of the PBP pathway in assimilation of the ribose moiety of nucleosides as well as the contribution of the resulting pyruvate to both energy and carbon metabolisms.

**Fig 3:**
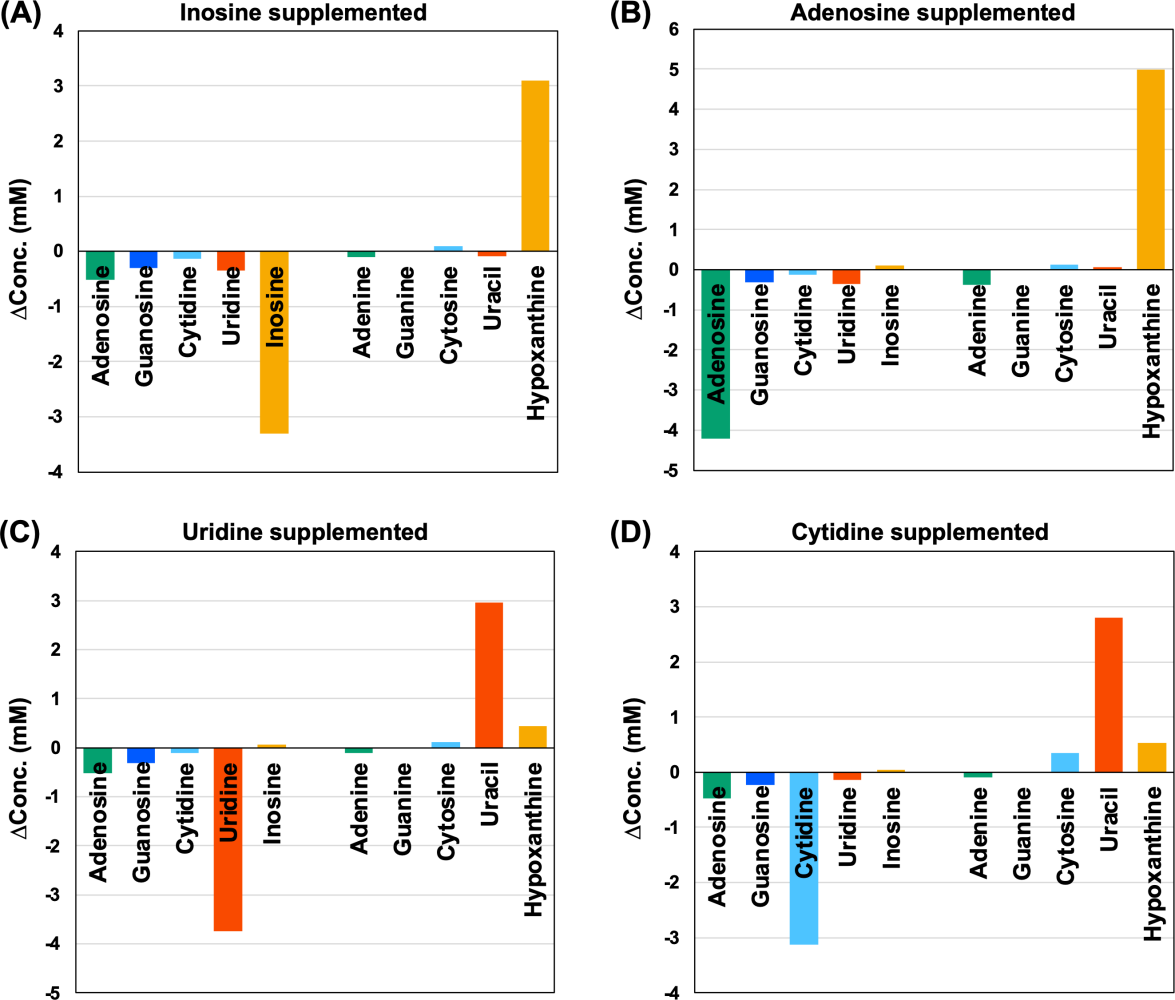
Changes in the concentrations of nucleosides and nucleobases in the culture supernatant during the growth of ALE22 in an ASW-YT medium supplemented with 0.1% (wt/vol) of inosine (**A**), adenosine (**B**), uridine (**C**), or cytidine (**D**). Cultivation was carried out at 85°C for 96 h (**A, C, D**) or 90 h (**B**). The concentrations of nucleosides and nucleobases before and after the cultivation were measured by LC-MS/MS.

The LC-MS/MS analysis of the culture supernatant also detected the accumulation of 5.6 mmol/L-culture of free nucleobases corresponding to the consumption of the total nucleosides, indicating that *T. kodakarensis* lacked the ability to degrade nucleobases. Interestingly, when adenosine was supplemented, hypoxanthine was detected instead of adenine (Fig. 3B). Supplementation of cytidine resulted in the accumulation of uracil (accounting for 89% of the consumed cytidine) along with a minor portion of cytosine (11%) (Fig. 3D). These findings demonstrated the presence of deamination activity toward adenine/adenosine and cytidine/cytosine in *T. kodakarensis*. In contrast, deamination of guanine was unlikely as xanthine was not detected when guanosine was consumed (data not shown).

### Re-sequencing analysis of the mutant

The whole genomes of the strain ALE22 and the parent strain KUW1 were re-sequenced to identify the mutations responsible for the nucleoside-dependent growth of the evolved strain. The comparison of the two genomes identified 12 mutations, which were five SNPs with nonsynonymous substitutions and four frameshift mutations in coding sequences, and three mutations in non-coding intergenic regions (Table 2). Neither large-scale deletion nor duplication was detected in the mutant genome.

**TABLE 2:** Mutations identified in ALE22 strain^*a*^

Gene	Annotation	Position	Nucleotide change	Codon change	Amino acid change
*tk0309*	Elongation factor EF-2	264125	T→C	AGG→GGG	R715G
*tk0634*	Hpt domain-containing protein	540909	G→GGA	Frameshift	E533fs
*tk0657*	BMP family ABC transporter substrate-binding protein	559628	C→T	ACG→ATG	T39M
*tk0657*	BMP family ABC transporter substrate-binding protein	559861	A→G	AAG→GAG	K117E
*tk0684*	Aromatic amino acid transport family protein	591594	∆A	Frameshift	S196fs
Intergenic	Downstream of *tk1107* (hypothetical protein) and *tk1108* (phosphoglucosamine mutase)	971852	C→CTT		
Intergenic	Upstream of *tk1186* (CBS domain-containing protein)	1042430	T→C		
*tk1514*	(d)CMP kinase	1333254	T→C	ATA→ATG	I127M
*tk1768*	Glycogen synthase	1570902	∆C	Frameshift	A380fs
*tk2076*	Formate dehydrogenase subunit alpha	1867724	∆G	Frameshift	T296fs
*tk2225*	Molybdenum cofactor biosynthesis protein A	2015577	G→T	AAC→AAA	N113K
Intergenic	Upstream of *tk2290* (form III ribulose-1,5-bisphosphate carboxylase/oxygenase)	2073434	A→G		

^*a*^ The mutation positions are based on the NCBI reference genome of *T. kodakarensis* KOD1 (NC_006624.1). “fs” indicates the amino acid position at which a frameshift mutation occurred.

### Contribution of overexpressed RuBisCO to the nucleoside-dependent growth

**Fig 4:**
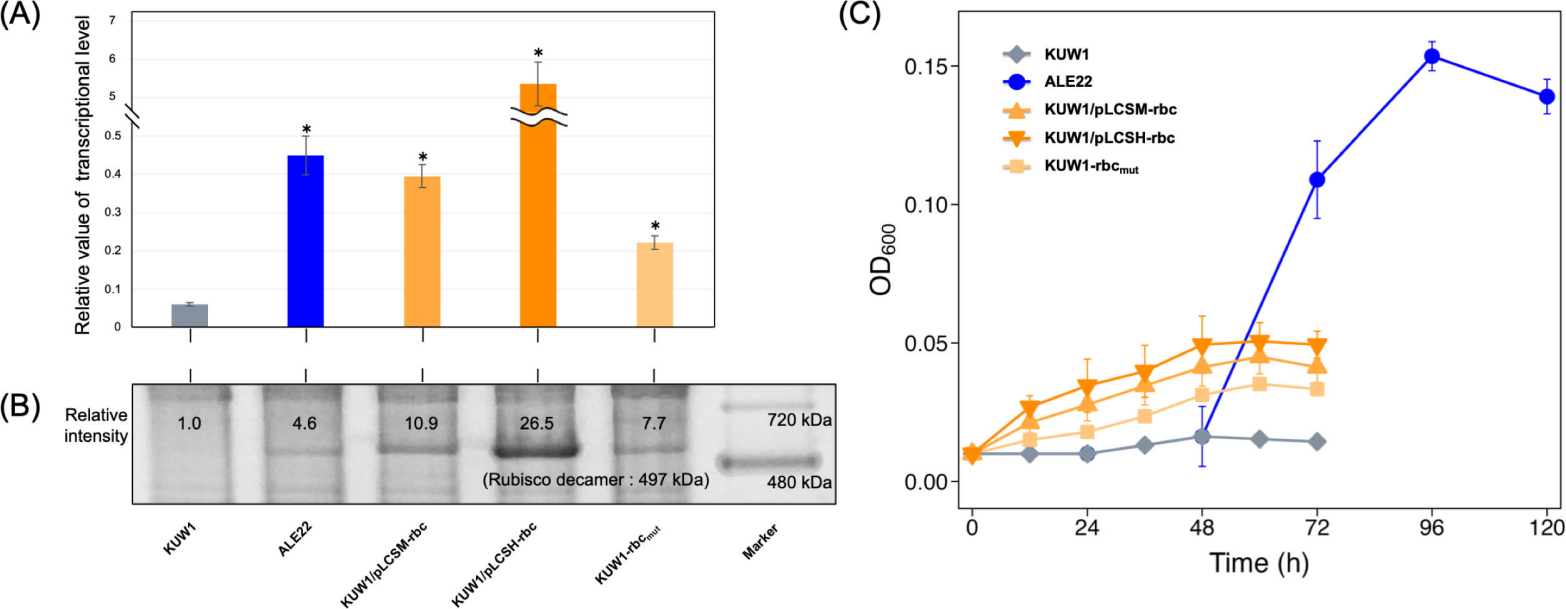
(**A**) Transcriptional analysis of *tk2290* for KUW1, ALE22, KUW1/pLCSM-rbc, KUW1/pLCSH-rbc, and KUW1-rbc_mut_. The cells were cultured in an ASW-YT-Pyr medium at 85°C for 16 h, and the total extracted RNAs were subjected to qRT-PCR analysis. The relative expression levels were calculated using the 2^−ΔΔCt^ method with *tk0308* as the housekeeping gene. Error bars represent standard deviations of technical triplicates (*P < 0.05 vs. KUW1). (**B**) Native-PAGE analysis of cell-free extracts from *T. kodakarensis* strains grown in an ASW-YT-Pyr medium at 85°C for 16 h. The relative intensity of the RuBisCO decamer band (497  kDa) was calculated relative to that of the control strain KUW1. (**C**) Growth properties of KUW1 (diamonds), ALE22 (circles), KUW1/pLCSM-rbc (triangles), KUW1/pLCSH-rbc (inverted triangles), and KUW1-rbc_mut_ (squares) in an ASW-YT medium supplemented with 0.2% (wt/vol) each of inosine and uridine (ASW-YT-IU). The cells were anaerobically cultivated in the 8 mL medium using a test tube at 85°C for 72 h or 120 h. Error bars represent standard deviations of three independent culture experiments except for the control strain KUW1.

The single-nucleotide substitution of A to G located 16-base upstream of the start codon of *tk2290* in ALE22 was presumed to affect the expression level of RuBisCO. Indeed, qRT-PCR analysis showed a 7.5-fold increase in the transcription level of *tk2290* in ALE22 when compared to that in the parent strain KUW1 (Fig. 4A). The overexpression of RuBisCO was also confirmed by protein electrophoresis. As the molecular mass of the RuBisCO subunit TK2290 (49.7 kDa) is similar to that of glutamate dehydrogenase (47.0 kDa), highly expressed in *T. kodakarensis*, Native-PAGE analysis was applied here. The non-denatured electrophoresis of the cell-free extract detected an approximately 500 kDa protein possibly corresponding to the decameric complex of RuBisCO (497 kDa) (33) in the evolved strain, but was not found in KUW1 (Fig. 4B).

While we observed the essential role of RuBisCO in the nucleoside-dependent growth of ALE22, as the strain ALE22-Δrbc, lacking the RuBisCO gene (*tk2290*), did not grow in ASW-YT with any nucleoside (data not shown). These results strongly suggested that higher activity of RuBisCO in the PBP pathway was one of the factors relevant to the nucleoside-dependent growth. The growth of the recombinant strains harboring a vector for the overexpression of *tk2290* was thus investigated. The strains KUW1/pLCSM-rbc and KUW1/pLCSH-rbc, harboring a vector for expression of the RuBisCO gene under the control of weak constitutive promoter (*pyrF* promoter) and strong constitutive promoter (*csg* promoter), showed 6.6- and 89-fold higher transcription levels, respectively, when compared with KUW1 (Fig. 4A). The results of Native-PAGE analysis also agreed with higher expression of RuBisCO in these strains (Fig. 4B). These RuBisCO-overexpressing strains actually showed growth upon the addition of certain kinds of nucleosides, such as inosine and uridine (ASW-YT-IU) as shown in Fig. 4C, demonstrating that higher expression of RuBisCO allowed *T. kodakarensis* to use nucleosides as growth substrates. We also constructed another recombinant strain by introducing the single A-to-G mutation at 16-base upstream of *tk2290* into the chromosome of the parent strain KUW1. The resulting strain KUW1-rbc_mut_ showed 3.7-fold higher transcription level of *tk2290* and 7.7-fold higher band intensity of the RuBisCO decamer in Native-PAGE than KUW1 (Fig. 4A and B). It was confirmed that the KUW1-rbc_mut_ strain was also able to grow in the ASW-YT-IU medium (Fig. 4C). The nucleoside-dependent growth of *T. kodakarensis* became possible by only one base mutation, although the growth ability was lower than that of the evolved strain ALE22.

### Contribution of the mutated nucleoside transporter to the nucleoside-dependent growth

**Fig 5:**
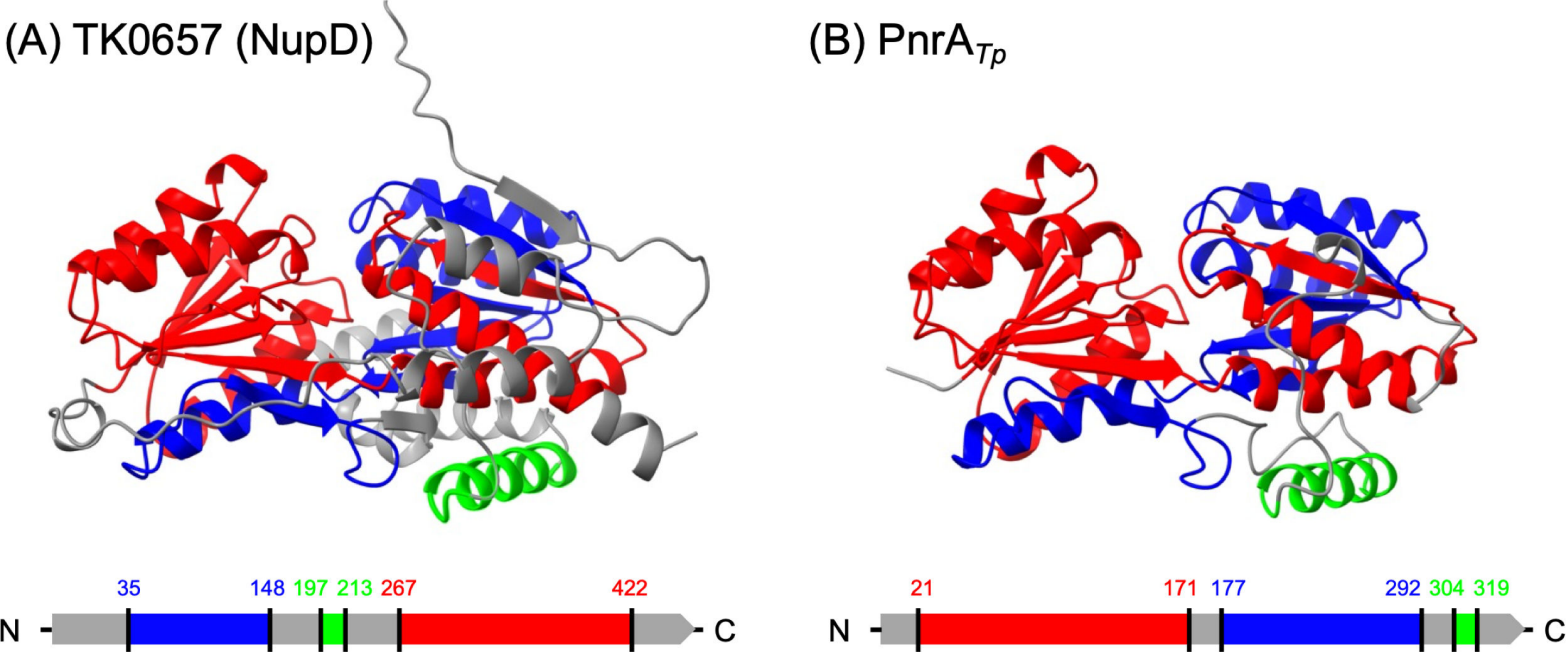
Structural models of *T. kodakarensis* TK0657 (NupD) predicted by AlphaFold3 (**A**) and *T. pallidum* PnrA*_Tp_* solved by crystal structure analysis (PDB ID: 2FQW) (**B**). The α-helices and β-sheets structurally conserved between TK0657 and PnrA*_Tp_* are highlighted in blue, red, and lime, respectively.

Other interesting mutations identified in the evolved strain ALE22 were two nucleotide replacements in *tk0657* corresponding to amino acid substitutions of Thr 39 by Met and Lys 117 by Glu in the translated product. Given that there is no apparent change in the expression level of *tk0657* as shown in Fig. S1, it is likely that these mutations are related to the function of TK0657. The gene cluster from *tk0657* to *tk0660* encodes a BMP family ABC-type transporter composed of periplasmic substrate-binding subunit TK0657, ATPase subunit TK0658, and permease subunits TK0659 and TK0660. It has been reported that an ABC-type transporter derived from *Lactococcus lactis*, BmpA-NupABC*_Ll_*, is a nucleoside-specific transporter, in which BmpA is a novel type of periplasmic substrate-binding subunit (34). TK0658-TK0659-TK0660 shared high amino acid sequence similarity with NupABC*_Ll_* (33%–47% identities in 85%–99% coverages), while TK0657 showed partial similarity of 35% identity within 36% coverage to BmpA*_Ll_*. Meanwhile, the AlphaFold3-predicted tertiary structure of TK0657 showed high similarity to two BmpA homologs, PnrA from *Treponema pallidum* (PDB 2FQW (35)) and that from *Streptococcus pneumoniae* (PDB 6Y9U (36)), with RMSD values of 0.731 Å for 714 atoms and 1.008 Å for 678 atoms, respectively. The acidic, basic, and aromatic amino acid residues within the substrate-binding pocket and the hinge-like conformation enclosing the substrate-binding site are highly conserved among the three proteins (Fig. S2). We hereafter designate TK0657 as NupD. Structural comparison between NupD and PnrA*_Tp_* revealed that the N- and C-terminal regions of NupD correspond to the C- and N-terminal regions of PnrA*_Tp_* (Fig. 5). Although NupD shares relatively low primary sequence similarity with PnrA*_Tp_* and PnrA*_Sp_* due to the flipped domain arrangement, the predicted folding of NupD closely resembles the previously determined structures of PnrA*_Tp_* and PnrA*_Sp_*, suggesting a functional correlation. The ABC-type transporter complex containing NupD was considered to be an archaeal variant of nucleoside-specific transporter, potentially involved in the nucleoside-dependent growth of the evolved strain.

**Fig 6:**
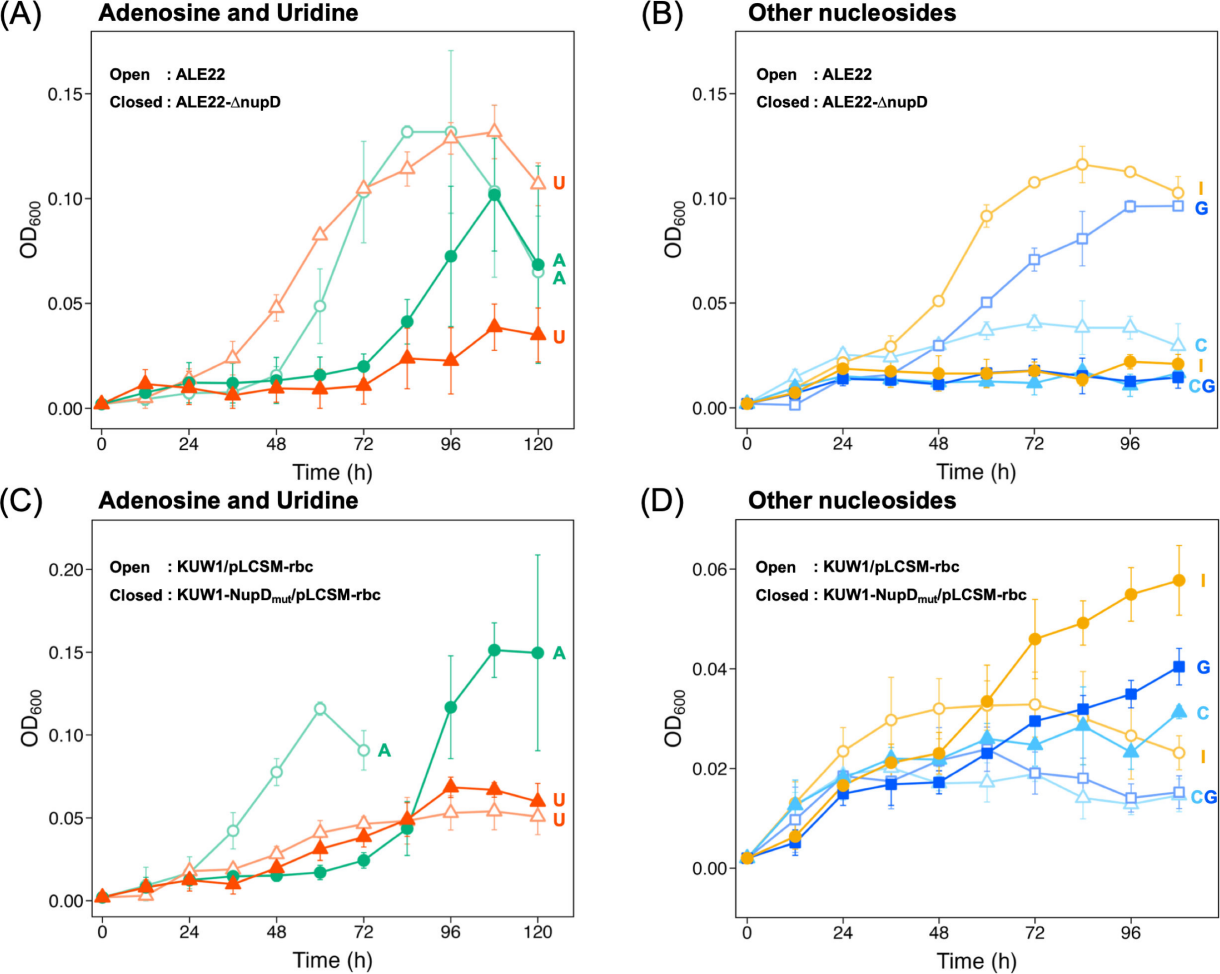
(**A**) Growth properties of ALE22 (light-colored line with open symbols) and ALE22-∆nupD (regular-colored line with closed symbols) in an ASW-YT medium supplemented with 0.2% (wt/vol) of adenosine (green circles) or uridine (red triangles), and (**B**) those in the medium supplemented with 0.2% (wt/vol) of guanosine (blue squares), inosine (yellow circles), or cytidine (sky-blue triangles). (**C**) Growth properties of the strains KUW1/pLCSM-rbc (light-colored line with open symbols) and KUW1-NupD_mut_/pLCSM-rbc (regular-colored line with closed symbols) in an ASW-YT medium supplemented with 0.2% (wt/vol) of adenosine (green circles) or uridine (orange triangles), and (**D**) those in the medium supplemented with 0.2% (wt/vol) of guanosine (blue squares), inosine (yellow circles), or cytidine (sky-blue triangles). The cells were anaerobically cultivated in the 8 mL medium using a test tube at 85°C for 120 h. Error bars represent standard deviations of three independent culture experiments.

The function of the mutated NupD in the evolved strain was investigated by gene disruption analysis. A new variant ALE22-∆nupD, an ALE22 derivative lacking the mutated *nupD*, showed delayed and slower growth in the adenosine- or uridine-supplemented media when compared with the parent strain ALE22 (Fig. 6A), and no growth in the guanosine-, inosine-, or cytidine-supplemented media (Fig. 6B). These results indicated the possible role of the mutated NupD in the uptake of nucleosides for the growth of ALE22. The above growth spectrum suggested that the transporter composed of the mutated NupD was likely the sole transporter for guanosine-, inosine-, and cytidine. It was also thought to contribute to the uptake of adenosine and uridine to some extent, while other transporter(s) may also play a role in this process. It should be noted that the two amino acid substitutions in NupD (T39M/K117E) were insufficient for the nucleoside-dependent growth of *T. kodakarensis*, as the reconstituted strain KUW1-NupD_mut_, having the corresponding mutations in the chromosomal *nupD*, did not grow in any nucleoside-supplemented media (data not shown). To further investigate the synergistic effects of RuBisCO overexpression and the *nupD* mutation, we compared the growth of KUW1 and KUW1-NupD_mut_ transformed with the RuBisCO-overexpression plasmid, pLCSM-rbc. The RuBisCO-overexpressing strain KUW1/pLCSM-rbc, showing partial growth in the ASW-YT-IU medium as described above, also grew in the media supplemented with adenosine, inosine, or uridine (Fig. 6C and D). It was further observed that the strain KUW1-NupD_mut_/pLCSM-rbc, harboring both the mutations in NupD and the RuBisCO-overexpression plasmid, additionally acquired the ability to grow in the guanosine- or cytidine-supplemented media and showed enhanced growth with inosine supplementation (Fig. 6D). This strain showed faster growth but an unexpectedly longer lag phase when compared with KUW1/pLCSM-rbc in the adenosine-supplemented medium (Fig. 6C). Although the reason has been unknown, the mutations in NupD may lead to excess uptake of adenosine and consequently have a negative impact on the growth of *T. kodakarensis*, as observed in the antimicrobial activity of adenosine against intestinal *Salmonella* (37).

## DISCUSSION

In *T. kodakarensis*, ribose moieties in nucleosides are converted to two molecules of pyruvate by the newly discovered carboxylating PBP pathway concomitant with CO_2_ fixation by RuBisCO (20–22). Although the pyruvate derived from the ribose moiety can act as an amino-acceptor in catabolic oxidation of amino acids, the wild-type strain of *T. kodakarensis* is incapable of growing in nutrient-rich media containing large amounts of peptides/amino acids and nucleic acids, unless supplemented with exogenous S^0^ or pyruvate. Nevertheless, the ALE22 mutant isolated in this study acquired the nucleoside-dependent growth ability in part by upregulated expression of RuBisCO and enhanced uptake ability for extracellular nucleosides. These results indicated that the carboxylating PBP pathway is not only important for the re-utilization of the pentose moieties in intracellular nucleic acids, but also potentially acts as a central metabolic module supporting cell growth on exogenous nucleosides along with peptides/amino acids. It was thought that nucleic acid-related compounds are available in natural environments where peptides/amino acids derived from other organisms are present. The nucleoside-dependent growth ability would be important for organisms preferring proteinaceous substrates, including Thermococcales hyperthermophiles, to inhabit such natural environments, although the wild strain of *T. kodakarensis* has lost the ability. Recent metagenomic analyses have detected the genes of form III RuBisCO and other enzymes involved in the carboxylating PBP pathway in bacteria belonging to candidate phyla radiation (CPR), a large group of uncultivated bacteria estimated to be an absolute symbiont with a minimal genome and dependency on the host for its nutrient acquirement (38, 39). These findings also suggest that the assimilation of exogenous nucleosides mediated by the PBP pathway has been important for some lineages of life.

A BMP family-ABC-type transporter composed of TK0657-TK0658-TK0659-TK0660, designated NupDABC, is the nucleoside-specific transporter, first identified in hyperthermophiles. The growth properties of the evolved strain ALE22 and its derivatives were consistent with the contribution of the mutated transporter to the nucleoside-dependent growth, facilitated by the enhanced uptake of various nucleosides. In the mutated NupD (TK0657), Lys117, located in the substrate-binding pocket, was substituted for the negatively charged Glu residue, which was expected to alter the affinity to nucleosides. However, docking simulations using AutoDock Vina revealed that the binding energies of five nucleosides to the predicted structures of NupD were not clearly altered by the mutation(s). The other mutation, substitution of Thr39 to Met located near the NupD-NupC interface, may affect the subunit interaction, and the consequent change in the overall folding of the transporter complex may enhance the transport efficiency.

Aono et al. reported that cytidine was not recognized by nucleoside phosphorylases to form R1P, unlike the other three types of nucleosides in *T. kodakarensis*, and proposed a possibility for the conversion of cytidine to uridine, followed by cleavage to uracil and R1P, and entry into the PBP pathway (20). We here observed markedly increased accumulation of uracil in the medium when cytidine was supplemented (Fig. 3D), strongly suggesting that most cytidine was actually deaminated to uridine and subsequently metabolized through the PBP pathway. This was also consistent with no cytidine kinase activity in the cell extract of *T. kodakarensis* despite the presence of TK1843, of which the recombinant enzyme accepted cytidine as a substrate for phosphorylation *in vitro* (20). As well, *T. kodakarensis* appeared to have high deamination activity toward adenosine (or adenine), as shown by accumulation of hypoxanthine with almost equivalent amount of adenosine consumed during the cultivation (Fig. 3). Although some kinds of bacteria, such as *Bacillus subtilis*, utilize the amino moiety in adenine as a nitrogen source (40), this would be unlikely for *T. kodakarensis* because amino moiety was sufficiently available during the growth on amino acids. The physiological role of the deamination of nucleobases in the hyperthermophile remains to be clarified.

The point mutation in the upstream region of *tk2290* markedly elevated the expression level of the form III RuBisCO (Fig. 4A). In *T. kodakarensis*, Thermococcales glycolytic regulator (Tgr) controls the expression of many genes encoding glycolytic and gluconeogenic enzymes by binding to the specifically recognized sequence called Thermococcales glycolytic motif (TGM). Although the RuBisCO gene (*tk2290*) was shown not to be under the control of Tgr (41), we found a TGM-like sequence (5′-ATTTACN_5_GTGAAA-3′, the bases matched with the consensus TGM sequence are underlined) at upstream of *tk2290*. The mutation in the evolved strain ALE22 was a substitution of the 5th adenine in the above sequence (16 base-upstream from the start codon of *tk2290*) by guanine, where the 5th adenine is highly conserved among known TGM sequences for the glycolytic/gluconeogenic genes regulated by Tgr (Fig. S3). It was presumed that the TGM-like sequence acted as an operator region for some transcriptional regulator, and the mutation led to a high transcriptional level for *tk2290*. In this case, it is hypothesized that a repressor other than Tgr was suppressing the expression, and that the mutation may have weakened its binding affinity, resulting in increased transcription.

The carboxylating PBP pathway would be a promising metabolism for the conversion of C_5_ sugars, because CO_2_ emission through this pathway was less than that by the conventional pentose phosphate pathway owing to the RuBisCO-mediated CO_2_ fixation, as shown in this study. Here, we demonstrated that the overexpression of RuBisCO and the mutation in NupD enabled the nucleoside-dependent growth for *T. kodakarensis*; the growth of the strain into which the mutations were reconstituted was much lower than that of the evolved strain. Further investigation is expected to identify unknown factors contributing to the nucleoside-dependent growth.

## SUPPLEMENTAL MATERIAL

Supplemental material aem.01712-25-s0001.pdf Table S1; Fig. S1 to S3.

ASM does not own the copyrights to Supplemental Material that may be linked to, or accessed through, an article. The authors have granted ASM a non-exclusive, world-wide license to publish the Supplemental Material files. Please contact the corresponding author directly for reuse.
