# Supplementary material for: Pentose bisphosphate pathway can act in central metabolism for nucleoside-dependent growth of Thermococcus kodakarensis strains
Source: Appl Environ Microbiol. 2025 Oct 29;91(11):e01712-25. doi: 10.1128/aem.01712-25 (PMC12628685; doi:10.1128/aem.01712-25)
Supplement: Supplemental material — Table S1; Fig. S1 to S3. [file aem.01712-25-s0001.pdf]

Appl. Environ. Microbiol.
10.1128/aem.01712-25

Pentose bisphosphate pathway can act in central metabolisms for nucleosides-dependent growth of *Thermococcus kodakarensis* strains

Tetsu Nishida, Yangzi Chen, Takehiro Azuma, Izumi Orita, and Toshiaki Fukui*

School of Life Science and Technology, Institute of Science Tokyo, Yokohama, Japan

*Corresponding author
Toshiaki Fukui
School of Life Science and Technology
Institute of Science Tokyo
B-37 4259 Nagatsuta, Midori-ku, Yokohama 226-8501, Japan
Tel/Fax: +81-45-924-5766
e-mail: tfukui@life.isct.ac.jp

Supplementary Information

Table S1
Fig. S1
Fig. S2
Fig. S3

Supplementary Table S1. Primers used in this study.

Name	Sequence (5′ >3′)	Note
Pcsg-5'	TATCGGCAAAAGGCGAATTAT	
Pcsg-3'	TCCACAACACCTCCTT	
TKglpD_Start-Up1000	AATCACCCTCGTGTCCGTGGGCTA	For construction of pUD3-up-P_{csg}-*glpFK*-down
TKglpD_Start-Down1000	CCTCTTCCTCAGCCTTGATTATGA	
TkglpD-Start-Inv5	TTCTTCCTTTTAGTTTGGAGGTGT	
TkglpD-Start-Inv3	CTGATTCTCCAGTTTTAAAGCTTT	
TMglpF-5'	ATGTCCGTGTACCTGGCCGAA	
TMglpK-3'	TGCCTCTTTTCATTTTGAATACAGCCTC	
tkRubisco-up-Fw	TGCTCTAGAACTCTCAAGGCCCTCGAGATGGACG	For construction of pUD3-ΔNupD and pUD3-rbc-up-mut
tkRubisco-down-Rv	TGCTCTAGAAGGCTCGTAAGAATGAAGGAAGAGG	
tkRubisco-inv-Fw	GGAAGATCTGCTGTTTCAGGGTTAGTGTTC	
tkRubisco-inv-Rv	GGAAGATCTGCCTTTCACCCCAGGTAAATTTAACG	
tkRubisco-RBS-mut-EcoR1-Rv	CGGAATTCAGCCGGTTATAACAACATCAACCATCG	
tk0657-up-Xba1-Fw	GCTCTAGATTGATAGCTTCAACCTGTCTTTCC	For construction of pUD3-Δrbc and pUD3-$NupD_{mut}$
tk0657-down-Xba1-Rv	GCTCTAGAGCCGGTTATGTCGACTCCATTGAGG	
tk0657-inv-Bgl2-Fw	GAAGATCTACCCTTTTCTTTTTCTCTTTCCTTGG	
tk0657-inv-Bgl2-Rv	GAAGATCTGGGAACACCCCTGAAAGGTTTGTC	
pLC71-inv2	GGCCTCATATGCATCACCTTTTTAACGGCCCTCTC	For construction of pLC71
pLC71-inv1DKanr	TACTAGTGCTAGCGCATGCCTTCTATCGCCTTCTTGACGAGTTCTTCTG	
tk2290-Rubisco-Nde1-Fw	GGAATTCCATATGGTTGAGAAGTTTGATACG	For construction of pLCSM-rbc and pLCSH-rbc
tk2290-Rubisco-Spe1-Rv	GGACTAGTTCAGACTGGAGTAACGTGACCCCAC	
qRT-TK0308-5'	GGCCAGCACCATCTTCCA	For qRT-PCR
qRT-TK0308-3'	GCTCGTGGTGCATCTCGAT	
qRT-Rubisco-Fw	CGACTACATGAAGGACGACGAGAAC	
qRT-Rubisco-Rv	TCGCGAACCATGTCTTCTTCTCAC	
qRT-tk0683-Fw	AGACTGCCATACTGGTGAACATAGC	
qRT-tk0683-Rv	GAACAGCTCCTCGTTGTAGTACGG	
qRT-tk1186-Fw	GGAGAAGTTCGGCAAAGGTAAAGTTG	
qRT-tk1186-Rv	CTCGTCGTTGACTATGGGTATCCTTG	
qRT-tk0657-Fw	CCAAGAGCGGTGAGTACGACATAATC	
qRT-tk0657-Rv	GTTGTGTGGCATTTCTGGATCGTAG	
qRT-fdh-Fw	CCAGAATGCTCGGAACCAACAAC	
qRT-fdh-Rv	TTCGCCTCTTCAATGTCCCTGTATG	

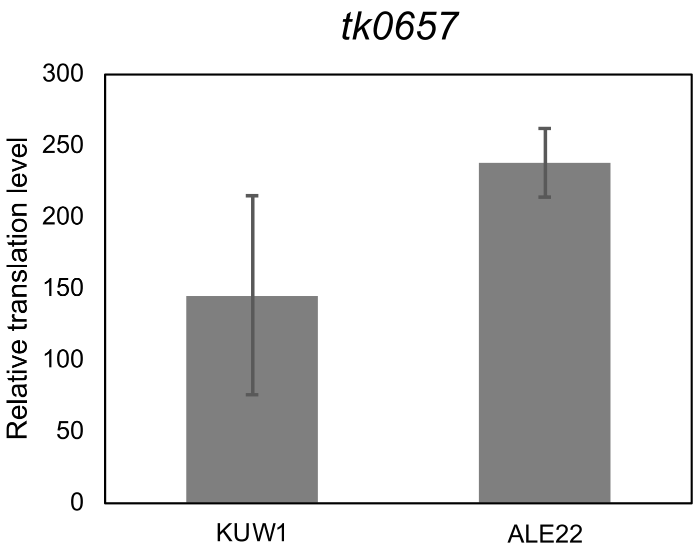

Supplementary FIG. S1. Transcriptional analysis of *tk0657* for *T. kodakarensis* strains KUW1 and ALE22. The cells were cultured in an ASW-YT-Pyr medium at 85˚C for 16 h, and the total RNAs extracted from the cells were subjected to qRT-PCR analysis. The relative expression levels were calculated using the $2^{-\Delta\Delta Ct}$ method with *tk0308* as the housekeeping gene. Error bars indicate the standard deviations of three technical replicates.

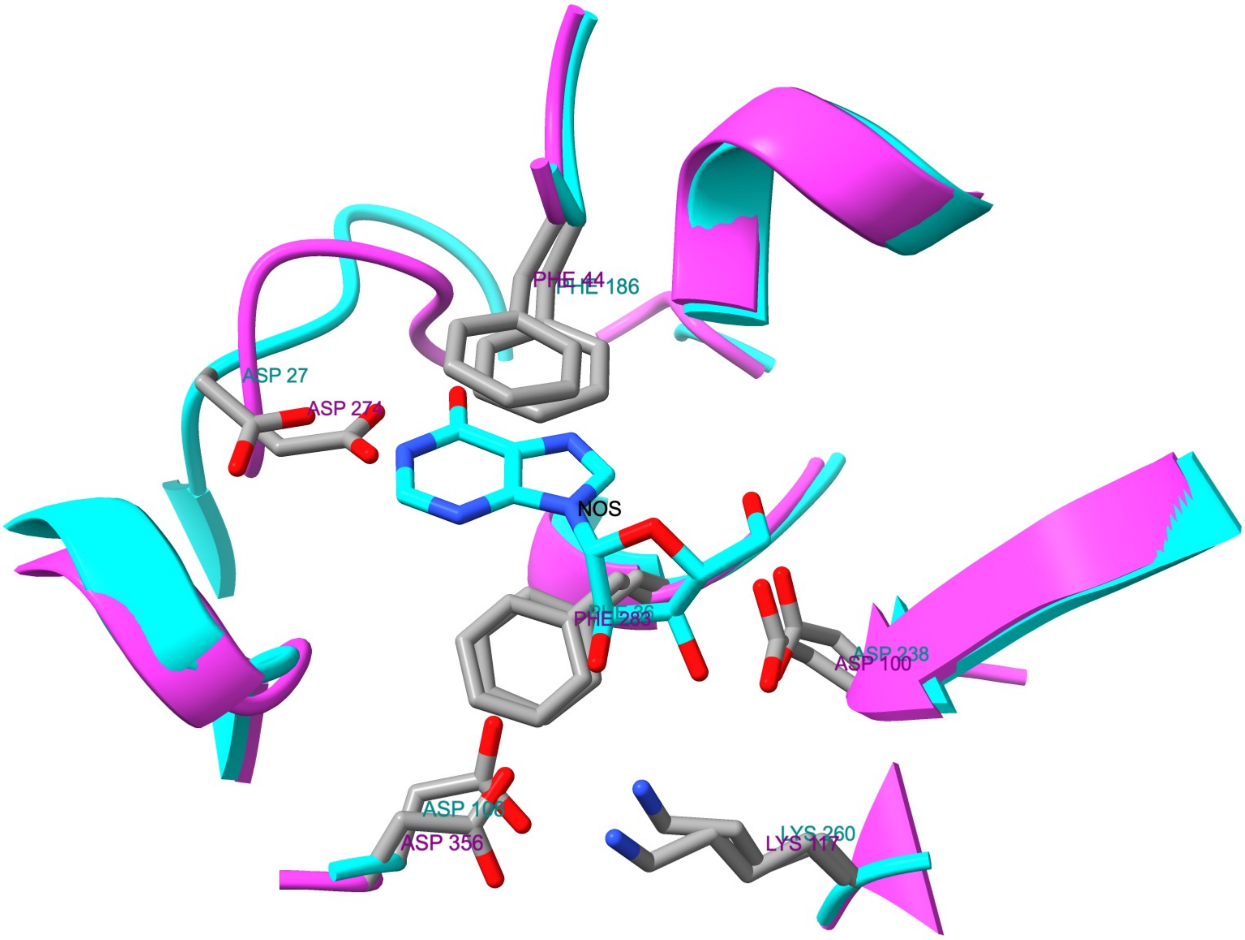

Supplementary FIG. S2. Tertiary structures of the substrate binding site in *T. kodakarensis* NupD predicted by AlphaFold3 (magenta) and that in *T. pallidum* PnrA determined by crystal structure analysis (cyan) (PDB ID: 2FQW). Acidic, basic, and aromatic amino acid residues within 10 Å from inosine (NOS) bound to the active site are shown as sticks.

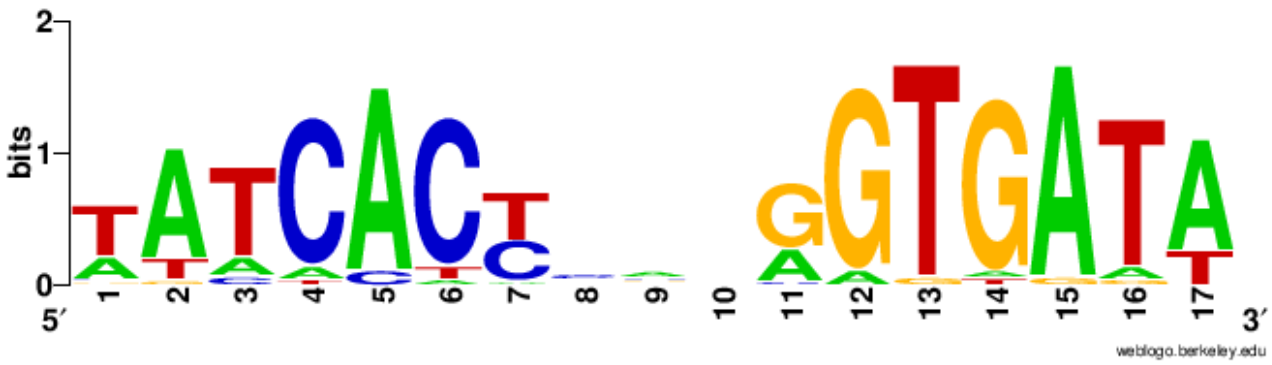

Supplementary FIG. S3. Sequence logo of consensus Thermococcales glycolytic motif (TGM) created from 25 genes regulated by Thermococcales glycolytic regulator (Tgr). The TGM sequences were extracted from the upstream regions of the following genes: *tk0376, tk0705, tk0866, tk0977, tk0989, tk1108, tk1110, tk1111, tk1136, tk1292, tk1295, tk1404, tk1406, tk1436, tk1622, tk1743, tk1771, tk1809, tk1884, tk2106, tk2129, tk2148, tk2163, tk2164,* and *tk2172*.
